# Fabrication and Characterization of Cu^2+^-Driven PTFE-Reinforced Artificial Muscle Polymer Membrane for Water Purification and Energy Harvesting Applications

**DOI:** 10.3390/membranes13090766

**Published:** 2023-08-29

**Authors:** Venkata Dinesh Avvari, P. S. Rama Sreekanth

**Affiliations:** School of Mechanical Engineering, VIT-AP University, Besides AP Secretariat, Amaravati 522237, Andhra Pradesh, India; venkatdinesh.22phd7081@vitap.ac.in

**Keywords:** Nafion, PTFE, reinforcement, water treatment, artificial muscle, energy harvester, triboelectricity

## Abstract

Ionic polymer membranes have not yet gained widespread practical application in areas such as water purification and energy harvesting due to their high cost and tendency to swell. The present study involved the fabrication of reinforced textile structures composed of polytetrafluoroethylene (PTFE)-reinforced Nafion membranes coated with non-precious metals, copper and silver, as a surface electrode by applying a chemical decomposition technique. Several mechanical, contact angle measurement and dielectric tests were conducted on membranes to evaluate their mechanical, wettability and conductivity properties. From scanning electron microscopy, it is clear that the formation of surface electrodes with uniform dispersion of metal particles. Scratch test reveals the adhesive strength between the coated metal particles and membrane. The silver-activated copper-coated membrane has a high contact angle of 121°. Thus, the fabricated membranes can have good antibacterial and adsorption properties for water treatment. The copper-coated membrane has a high Young’s modulus of 779 ± 80 MPa and a tensile strength of 29.1 ± 8 MPa, whereas the elongation at break is more for silver-activated copper-coated samples recorded as 158 ± 4%. The viscoelastic behavior of the membranes was analyzed through dynamic mechanical analysis (DMA). A sharp rise in the storage modulus (E′) value of 4.8 × 10^10^ Pa at ~80 °C at a frequency of 1 Hz on metal surface electrodes signifies an improvement in the strength of the material in comparison to the initial pure membrane. The successful enhancement of conductivity on the membrane surface via chemical deposition on the silver-activated membrane is 1 × 10^−4^ (S/cm) and holds great potential for facilitating voltage transmission through the tribolayer in the nanogenerators.

## 1. Introduction

The utilization and exploration of polymers in various fields, such as batteries [1], robotics, bio-engineering [2], sensors [3], water and air purification [4], and semiconductors [5], have been expanding due to their desirable properties including reversibility, chemical stability, and biocompatibility. Ionic polymers are a type of electroactive polymers that respond to applied electric voltage. Ionic polymer metal composites (IPMCs) are gaining popularity due to their unique characteristics, such as flexibility, lightweight, moldability into various shapes, low-voltage requirement, and efficient bending actuation. IPMC has the capability to function as both a sensor and an actuator [6]. The application of a time-varying electric field across the surface electrodes (noble and non-noble metal) of an IPMC will result in its deformation. Furthermore, the bending deformation is influenced by both the magnitude and direction of the applied electric potential, as well as the moisture content of the base polymer. When an IPMC undergoes mechanical deformation, it develops a density differential within the material. The cations in a hydrated state exhibit a tendency to migrate from regions of higher density to those of lower density. Thus, the generation of an electrical charge is facilitated, subsequently enabling its conduction through the electrodes to be harvested [7].

Furthermore, the cation-exchange polymer-based membrane technology is extensively utilized in filtration and electrolyte membrane fuel cells for the production of energy through cleaning waste water simultaneously. Nevertheless, a significant proportion of these entities encounter mechanical and thermal breakdowns when subjected to certain operational circumstances [8]. Certain materials may exhibit burn, shrinkage, and structural failure when operating under conditions of low relative humidity and high temperatures as a consequence of internal defects such as tearing, cracking, and pinholes. Consequently, enhancing these materials’ thermal stability, chemical durability, and mechanical robustness is imperative to provide them appropriate for deployment in diverse environments. Additionally, the negative charge carrier polymer has the advantage of adsorption of heavy metal ions from wastewater, which is protecting human health and the environment is of the utmost significance. This can be done through a range of techniques, such as ion exchange, bio-sorption, adsorption, and reverse osmosis. Hence, materials that possess porous and micro/nanostructured characteristics, resulting in a substantial increase in surface area, are extensively employed for their potential adsorption capabilities [9].

PFSA-based polymers, such as Nafion^®^, a well-known artificial muscle polymer, and sulfonated tetrafluoroethylene-based fluoropolymer, exhibit exceptional thermomechanical and electrical characteristics. It is a copolymer of perfluoro-3,6-dioxa-4-methyl-7octene-sulfonic acid and tetrafluoroethylene (Teflon™) that has both amorphous and semi-crystalline regions, in addition to hydrophilic sulfonic acid moieties. The backbone chains provide stabilization to the initial segment of the structure. The subsequent segment of the chain is distinguished by the existence of lateral branches and sulfonic acid moieties. The region that facilitates proton conduction is constituted by hydrophilic sulfonic groups, which make it well-suited for a variety of advanced uses such as energy production, purification, separation, actuation, fuel cells, and chemical sensors [10]. Furthermore, to decrease the expenses associated with production, the utilization of non-precious metals has been suggested as a viable option for manufacturing IPMC materials. R.K. Jain et al. [11] constructed a sensor utilizing a Nafion 117 strip coated with gold to detect EMG signals generated from the index finger of a human being. The sensor was integrated with a proportional integral derivative control system to monitor muscle contraction or expansion.

Therefore, in order to reduce the cost of electrode coating, Karan Surana et al. [12] used polyaniline and graphene as an electrode on Nafion film. The conductivity of the membrane proportionally increases with the percentage of graphene due to the molecular and ionic interactions. Furthermore, the film coated with graphene exhibited the highest proton conductivity, with a percentage of nearly 90% and a dielectric constant that can be used for energy storage. Energy harvesting from IPMC under mechanical vibration of a tapered film was done through finite element analysis. Under the 5 Hz, the maximum voltage output obtained from the taper shape film is 38 mV, and 34 mV from uniform beam shape at 22 Hz [13].

The high-performance artificial muscle incurs significant costs due to the utilization of platinum or gold for the metal electrodes. It is expected that alterations in processing and operational parameters could potentially decrease the dependence on these metallic elements. The arrest of moisture presents a significant limitation that can influence the experimental performance. The research aim is to investigate the potential of utilizing copper (Cu) as a surface electrode to enhance the performance of IPMC towards water treatment and energy harvesting, particularly due to its non-precious metal properties rendering it a viable option for implementation as metal ion adsorption for water purification. The proposed IPMC is fabricated using the chemical deposition method. The present technique for producing copper-electrode IPMCs involved multiple iterations of trial-and-error in order to optimize the process parameters. The current investigation endeavors to attain the objectives of the preparation and characterization of the mechanical and electrical properties of the Cu-IPMC and a comparative analysis of the performance of Cu-IPMC with respect to Cu and Ag surface activation.

## 2. Materials and Methods

### 2.1. Materials

A Teflon Fabric-Reinforced Nafion (TFRN) membrane of thickness 320 μm was procured from the Ion Power Nafion™ store, 954 Washington Ave., Tyrone, PA 16686, USA. It exhibits two prominent characteristics, namely reduced edge leakage and enhanced expansion uniformity. Silver nitrate (AgNO_3_) was purchased from Fisher Scientific Co, dextrose anhydrous (C_6_H_12_O_6_), ammonia solution (NH_3_), sodium hydroxide (NaOH), hydrochloric acid (HCl) 3 mol/L solution, cupric sulfate pentahydrate 99.5%, 2,2-Bipyridyl, EDTA disodium salt dihydrate, formaldehyde solution and lithium chloride were purchased from Sisco research laboratories Pvt. Ltd., Mumbai, India. All chemicals utilized in the research are in original form without undergoing additional purification procedures.

#### Fabrication of Cu-IPMC by Chemical Deposition Technique

**Figure 1:**
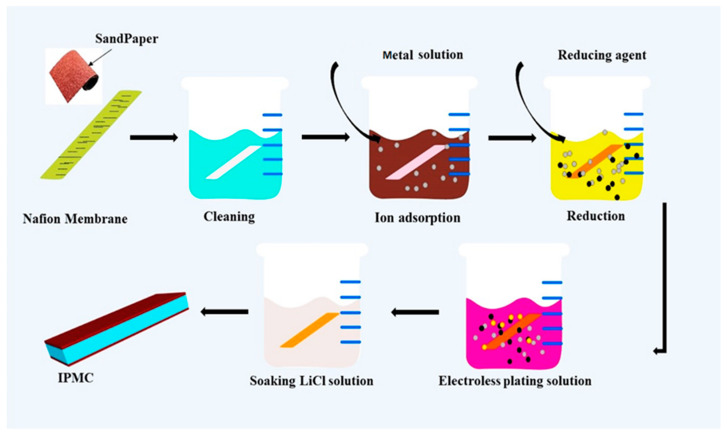
Electroless plating process of copper on TFRN membrane.

The state-of-the-art fabricating process of ionic polymer metal composite (IPMC) can be achieved through the utilization of chemical plating [14], which is considered to be a straightforward technique. The fabrication process is shown in Figure 1 and describes each step below.

Prior to electroless plating treatment, the membrane’s surfaces are roughened through the use of sandpaper with a grain fineness of P1200. This procedure enhances the adhesive strength and surface area to facilitate surface activation. The membrane, which had been roughened, underwent a cleaning process with the use of distilled water. It was subsequently immersed in a hydrochloric acid solution that had been heated to a high temperature for a duration of 45 min. This step was carried out to eliminate any impurities and silica particles that had become lodged within the membrane. The membrane was then rinsed with deionized water in order to remove any residual acid and facilitate swelling.

The process of ion adsorption involved immersing pre-treated membranes in a solution of silver nitrate solution prepared in an amber-colored bottle (AgNO_3_, 10 g/L, 100 mL) for a duration of 6 h to activate the surface of the membranes.

Copper plating solution: This step is crucial in enhancing the thickness of the surface electrode while reducing the surface resistivity. Following a DI water rinse, the membrane is immersed in a plating solution bath with the mentioned concentrations in Table 1 at 50 °C for 30 min.

**Figure 2:**
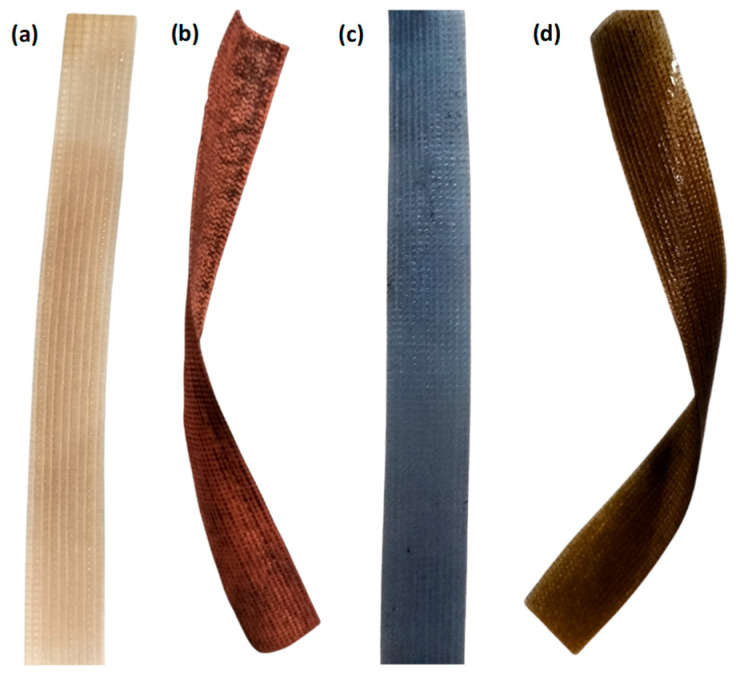
(**a**) Pure membrane, (**b**) copper-activated copper-coated membrane (Cu-Cu), (**c**) copper-activated silver-coated membrane (Cu-Ag), and (**d**) silver-activated copper-coated membrane (Ag-Cu).

Ion exchange is performed by immersing the Cu-IPMC sample in a lithium chloride solution (LiCl) for a duration of 60 min. This process facilitated the replacement of H^+^ ions with Li^+^ ions. Ultimately, the specimens are conserved in deionized water for characterization. Similarly, two more samples are prepared with the same procedure; however, at the ion adsorption stage, CuSO_4_·5H_2_O is used for both the samples for surface activation and silver-plating solution with dextrose anhydrous as a reducing agent and copper plating solutions taken for electroless plating solutions, respectively. Therefore, the laminated Cu-IPMC has a PTFE-reinforced ionomer membrane layer as an intermediate layer and a surface electrode layer, as shown in Figure 2.

### 2.2. Methods

#### 2.2.1. Microscopy Study

Optical microscopy on ZEISS was used to analyze the membrane structures at the microlevel, and SEM study on CARL ZEISS EVO10 equipment (Carl Zeiss Microscopy, LLC, White Plains, NY, USA) was used to analyze the morphology and microstructure of all specimens. Gold sputter coating was applied to the surface of the membrane sample in order to achieve the desired level of conductivity before observation. Subsequently, the specimen was affixed onto the sample holder and subjected to examination at a magnification level of 5000×, utilizing an applied voltage of 10 kV.

#### 2.2.2. Mechanical Characterizations

In order to determine the ultimate tensile strength, Young’s modulus, and membrane elongation, specimens were prepared in accordance with the ASTM D882 standard. The specimens acquired were subjected to tensile testing using a Tinus Olsen 10 KL computerized universal testing machine, which has a maximum load capacity of 10 kN. The tests were conducted within a temperature range of 22–25 °C.

The experiment was conducted at a crosshead speed of 2 mm/min. Three samples of each composition were subjected to testing to ensure precision and minimize inaccuracies, and the resulting mean values were recorded.

#### 2.2.3. Surface-Wetting Characterization

The contact-angle was measured using HOLMARC Opt Mechatronics Pvt. Ltd., Kochi, India. The interaction of water and a surface defines the porosity of the material. The contact angle is the geometric angle created at the water–air–solid interaction. This angle indicates water wetting. Low contact angle values indicate a surface’s affinity for water, whereas high values show a tendency to resist water. Surface-wetting characterization is done using sessile-drop goniometry due to its simplicity [15].

#### 2.2.4. Scratch Test

The utilization of a destructive testing methodology (scratch testing) was performed for the evaluation of the adhesive characteristics of metal coatings on membranes. The scratch test was performed on a DUCOM Scratch tester TR-101-IAS, manufactured in Tokyo, Japan. The scratch velocity was recorded as 10 mm per minute, whereas the loading rate was measured at 1 N.

#### 2.2.5. Dynamic Mechanical Analysis

A dynamic mechanical analyzer, SII Nanotechnology/DMS6100 Tester, Chiba, Japan, was utilized to perform thermodynamic analysis. The frequency was held constant at 1 Hz while the temperature spanned from 30 °C to 220 °C. The temperature range was utilized to assess the loss and storage modulus as well as the internal friction, represented by tan(δ).

#### 2.2.6. Fourier Transform Infrared Spectroscopy (FTIR)

The fabricated IPMC membranes were subjected to FTIR analysis using an Agilent technologies Cary 630 model FTIR device, Santa Clara, CA, USA. A total of 32 spectra were accumulated within the wavelength range of 400–4000 cm^−1^ to facilitate the identification and comparison of different functional groups and phases. The measurement of consolidated surfaces was conducted utilizing attenuated total reflectance mode.

#### 2.2.7. X-ray Diffraction (XRD)

X-ray diffraction (XRD) was performed on a MiniFlex diffractometer (Rigaku, Tokyo, Japan) using Cu Kα radiation over a 2θ range from 10° to 90° to measure the Phase analysis of IPMC.

#### 2.2.8. Electric Properties

The dielectric properties investigation was conducted utilizing an HIOKI low-frequency LCR meter (IM 3533-01) and an HIOKI High-frequency Impedance Analyzer (IM 7581) within the frequency spectrum of 1 Hz to 200 kHz. A specimen was employed to bridge the electrodes and subsequently placed inside the dry temperature calibrator (DPI-1100). Subsequently, the dielectric characteristics of the specimens were evaluated at an ambient temperature of 25 °C.

The electrical energy storage and dissipation of materials are influenced by their dielectric characteristics, which are dependent on both frequency and temperature. The relative permittivity (ε′), dielectric loss (ε″), conductivity (σ), and tan δ of fabricated IPMC were calculated as per reference [16].

## 3. Results and Discussion

### 3.1. Morphology and Microstructure

**Figure 3:**
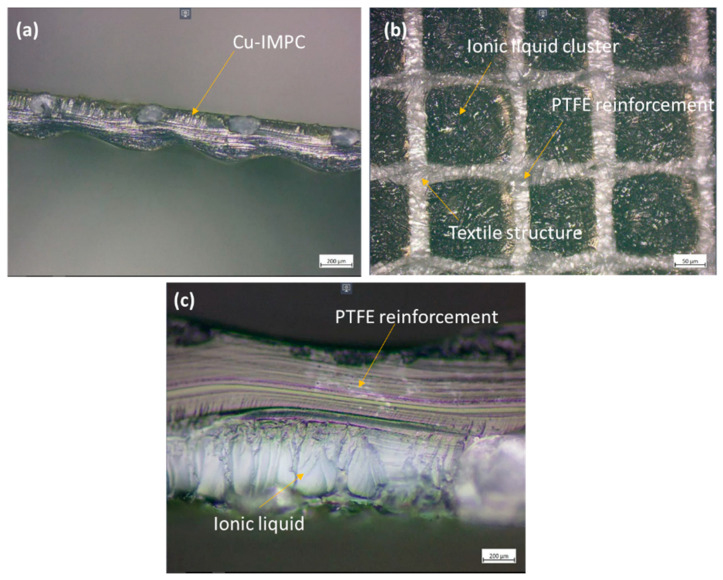

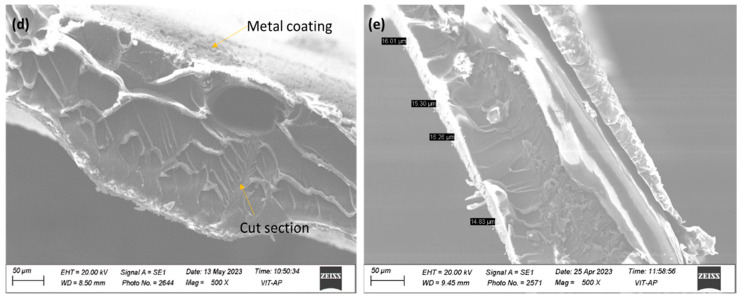
(**a**) Cu-IPMC, (**b**) microstructure of pure PTFE-reinforced Nafion, (**c**) presence of ionic liquid with reinforcement, (**d**) SEM of electrode layer and cut section of PTFE, and (**e**) electrode thickness.

The examination of surface morphology features of PTFE-reinforced Nafion thin film before and after modifications was conducted through a microscope and scanning electron microscopy (SEM), emphasized in Figure 3. A characteristic of textile-like pattern arresting ionic liquid clusters and ionic liquid with PTFE as a backbone is clearly seen in the microstructure, Figure 3a–c. The sample consists of an electrode layer of thickness 15.6 µm with silver and copper particles that are evenly distributed with diameters ranging in the tens of nanometers as shown in Figure 2 and Figure 3d,e. Despite the uniform distribution of particles on the surface, the chemical plating process results in the formation of small micropores. It is possible that there is a lack of enough adhesion between the surface layer of the membrane and copper particles. However, the presence of pores on the membrane may confer an additional benefit to the antibacterial efficacy of water purification, as it enables the entrapment of microorganisms and the adsorption of metal ions [17]. Moreover, in the context of nanogenerator implementation, a surface that is coated with Cu and Ag exhibits uniformity and is free of irregularities, indicating a desirable level of adhesion between the Nafion film and the metal particles. Consequently, the facilitation of current flow through the IPMCs is enabled for the purpose of energy generation.

### 3.2. Fourier Transform Infrared Spectroscopy (FTIR)

**Figure 4:**
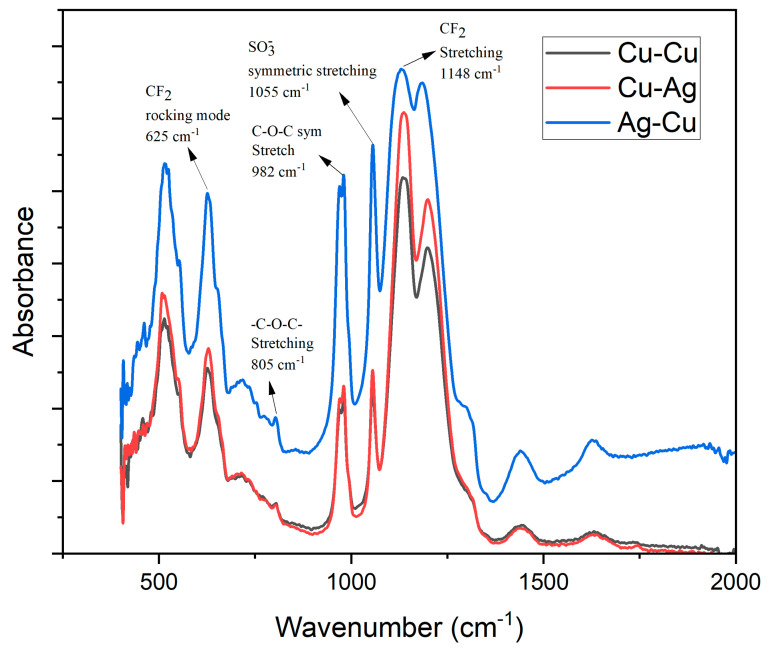
FITR analysis of Cu-Cu, Cu-Ag, and Ag-Cu IPMC membranes.

The FTIR spectra are illustrated in Figure 4. This study concerns the allocation of vibrational bands in Nafion. The primary absorption bands and their corresponding assignments are as follows: The PTFE membrane exhibits absorption peaks at 1200 and 1148 cm^−1^, which can be attributed to the stretching vibration of C-F. The absorption spectrum of Nafion exhibits distinct peaks at 982 cm^−1^ and 1055 cm^−1^. These peaks correspond to the stretching vibration of C-O-C on the side-chain of Nafion, as well as the symmetrical stretching vibration of −S$O_{3}^{-}$ and C-C, respectively [18].

### 3.3. Mechanical Properties

**Figure 5:**
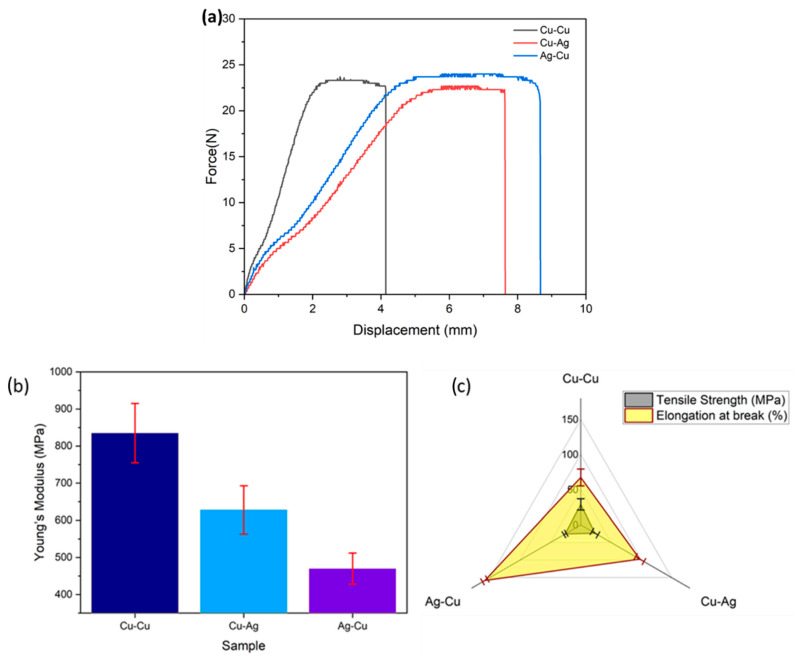
(**a**) Force-displacement curve, (**b**) Young’s modulus, and (**c**) tensile strength and elongation at break.

In order to obtain a more comprehensive assessment of the electrode’s quality, an IPMC underwent a stress-strain analysis. Figure 5a–c demonstrates the force-displacement curves, Young’s modulus, and elongation strain of Nafion membranes coated with copper and silver, and their corresponding results are shown in Table 1.

The enhancement of strength in the polymer is attributed to its crystalline phase, which is characterized by more significant intermolecular bonding [19]. Thus, in addition to the presence of crystallinity in Nafion, Figure 5a demonstrates that the incorporation of copper and silver through electroless plating into Nafion membranes enhanced their strength by 50% in comparison with the pure membrane [20]. This can be attributed to the strong adhesion between the metal particles and the membrane surface, which is facilitated by the attraction between positive and negative charged ions.

Likewise, the elastic modulus of a polymer is considered a fundamental mechanical property. It demonstrates a significant reliance on the material’s structure and manufacturing methodology. Due to the fact that copper has a higher Young’s modulus than silver, a significant number of copper particles were deposited on the surface of the Cu-Cu membrane during the chemical plating procedure. Figure 5b demonstrates that the copper surface-activated copper-coated IPMC membrane had a larger modulus owing to its hardness than the other two samples, Ag-Cu and Cu-Ag membranes, by 40% and 20%, respectively.

However, in the case of the elongation at break, surface activation metal ions played a major role because silver particles would strongly lock to the surface of the Nafion membrane compared with copper. The % of elongation at break was more than double for Ag-Cu IPMC compared with Cu-Cu film, as shown in Figure 5c and Table 1. Thus, the utilization of surface electrodes in the fabrication of IPMCs can result in the increase of crystallinity and improved mechanical properties, making them effective for energy production in both a piezoelectric and triboelectric nanogenerator [21].

### 3.4. Contact Angle Measurement

**Figure 6:**
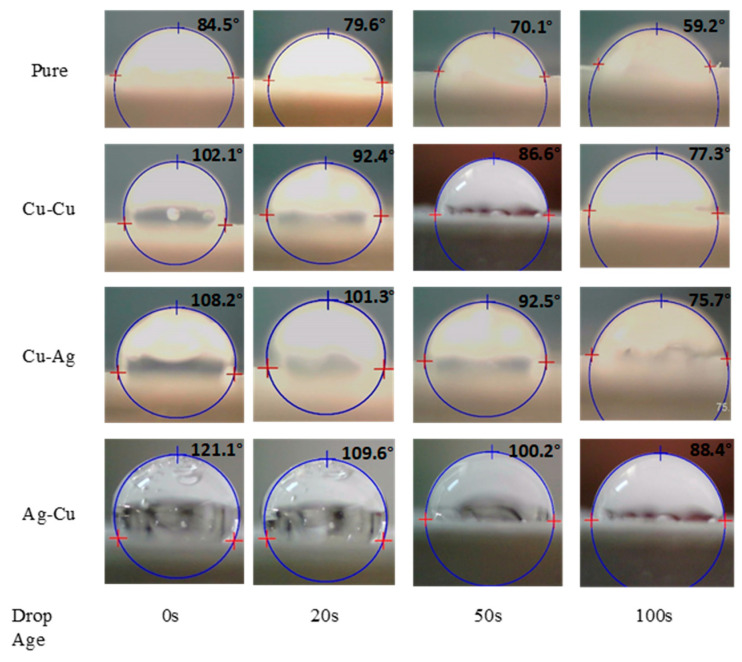
Contact angle measurements on pure and chemical-plated polymer membranes.

The contact angle data for water on the Nafion membrane at various time intervals is illustrated in Figure 6. The images of the reduction in water contact angle were captured and analyzed across all the samples at various time intervals. The reduced wetting by water is characterized by a tetrafluoroethylene backbone that creates a cohesive hydrophobic phase enveloping the hydrophilic domains that are constituted by the sulfonic acid side chains [22]. The sessile drop initially penetrates the surface of the Nafion membrane, causing rapid swelling of both its hydrophilic and hydrophobic domains due to the polarity of water. As a result, the contact angle decreases to 59.2° on the pure membrane.

However, the Ag-Cu membrane shows a higher contact angle at the start and ends at 88.4º, which is almost 20% higher than the Cu-Cu and Cu-Ag. In addition, the rapid swelling behavior of water on electroless process metal-coated membranes is absent due to the presence of metal particles covering the membrane surface. The adhesion energy of the water/metal system is expected to be influenced by the wettability of metals, which in turn is reliant upon the charged particles present on the substrate surface. The wetting behavior is influenced by the varying free electron densities [23] of metallic substrates.

Furthermore, the anticipated extent of membrane swelling is restricted by the reinforcement of polymer and the observed absorption and wetting characteristics, taking into account the duration of time on coated membranes. The adsorbent characteristic of the fabricated membrane involves the attachment of functional groups onto the surface and pore wall electrostatic force of polymer membranes. Hence, it is possible to eliminate various heavy metal ions, such as Zn(II), Pb(II), and Cr(VI), from wastewater through the utilization of adsorbents based on ionic polymers [24].

### 3.5. X-ray Diffraction (XRD) Analysis

**Figure 7:**
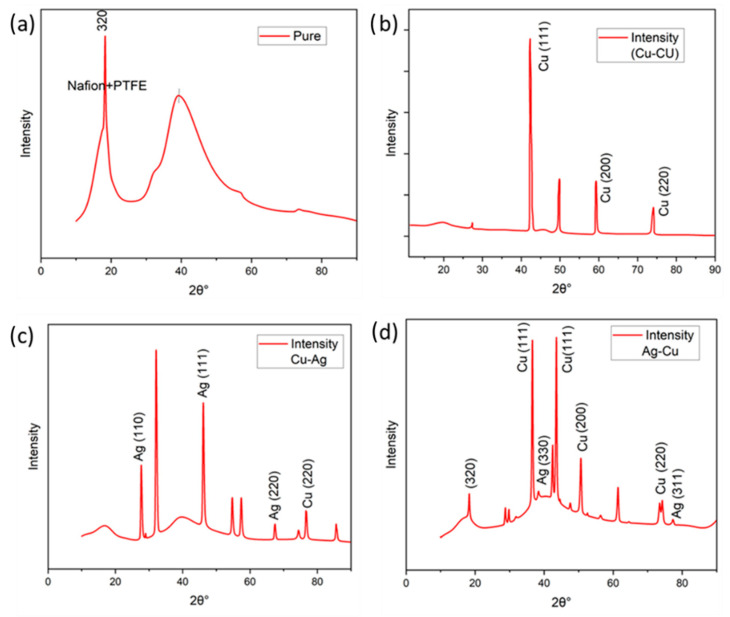
XRD analysis of (**a**) pure, (**b**) Cu-Cu, (**c**) Cu-Ag, and (**d**) Ag-Cu.

The crystal structures of IPMC Nafion membranes were examined through the use of X-ray diffraction (XRD) analyses. Figure 7. displays the X-ray diffraction (XRD) results of the Nafion membranes that were fabricated through electroless plating. The experimental data suggests that the highest peak observed within the 10–20 range can be segregated into two separate peaks via the process of deconvolution. The diffraction pattern obtained from the membranes exhibits a distinct peak at 2θ ≈ 17.5, which signifies the presence of crystalline regions in the membrane, which is more important in energy production [18], as depicted in Figure 7a. Furthermore, the presence of copper and silver elements was identified based on their respective material peaks through this analysis, as represented in Figure 7b–d, which would help to increase the crystallinity and surface conductivity for nanogenerator preparation [21].

### 3.6. Scratch Test Analysis

**Figure 8:**
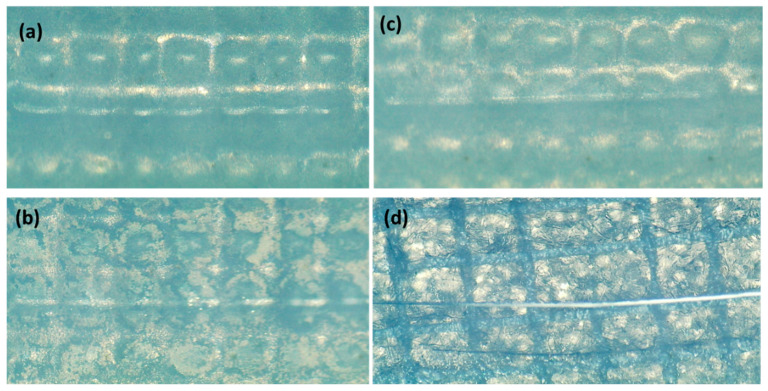
Scratch test of (**a**) pure membrane, (**b**) copper-activated copper-coated membrane (Cu-Cu), (**c**) copper-activated silver-coated membrane (Cu-Ag), and (**d**) silver-activated copper-coated membrane (Ag-Cu).

The performance of the Nafion membrane is influenced by various factors, including surface roughness, surface adsorption, and the presence of a hydrophilic negatively charged sulfonate group [25]. The utilization of a roughened substrate membrane has been found to enhance the adhesive strength between the electrode layer and the substrate, hence preventing the detachment of the metal electrode. The adhesive strength of interlayer films of Ag, Cu, and PTFE-reinforced Nafion membranes was measured using the scratch test technique at a load of 1 N. The scratch test is well-recognized as a reliable and effective technique for evaluating the quality of coated surfaces [25]. Figure 8 shows the surface membrane abrasions of all the samples and, as the results are shown in Table 2, Ag-Cu > Cu-Ag > Cu-Cu coating strength. The adhesive coating strength was assessed through the following equation [26]:

$$P=\frac{F_{max}-F_{avg}}{S}$$

where *P* is the adhesive strength, *F_max_* is the maximum lateral force during the debonding process, *F_avg_* is the average lateral force exerted by the indenter, and *S* is the scratch area. Furthermore, In the scratch test, the coefficient of friction (CoF) shows cracking or delamination of coating on the film with the sudden increment or decrement of CoF value. It is clear from the results shown in Table 3, the Ag-Cu has the lowest coefficient of friction, indicating a strong adhesion bond has formed between the metal and polymer layers.

### 3.7. Dynamic Mechanical Analysis (DMA)

**Figure 9:**
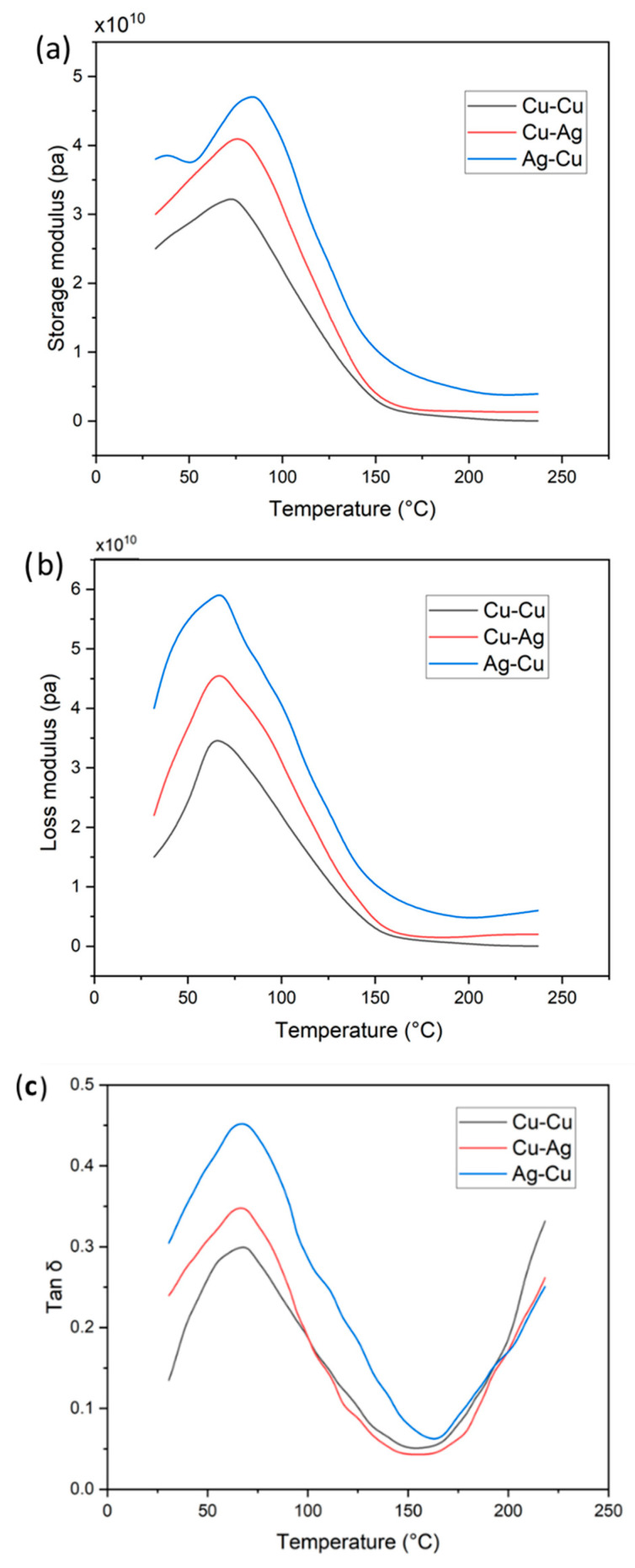
DMA analysis of (**a**) Cu-Cu, (**b**) Cu-Ag, and (**c**) Ag-Cu.

DMA results of all the samples are shown in Figure 9a–c and are compared with pure PTFE-reinforced Nafion [25]. For the silver-activated copper-coated Nafion, storage modulus (E′) increases to 4.8 × 10^10^ Pa at ~80 °C at a frequency of 1 Hz, which is higher than the pure [27] and Cu-Cu and Cu-Ag membranes. Additionally, a little peak shift is seen in Figure 8a with temperature increase; this shift is dependent on the strength of the interlocking bonds between the metal ions and membrane chain length. The data presented in Figure 8b indicates that the loss modulus (E″) of IPMC membranes exhibits a noticeable rise in response to an increase in temperature (60 to 100 °C), which is attributed to the occurrence of chain flow resulting from a significant expansion in volume. Consequently, compared with other membranes, Ag-Cu IPMC possesses a higher loss modulus of 5.95 × 10^10^, owing to the viscous nature of the material. The results suggest that the copper coating with silver activation exhibits a higher level of internal storage energy, indicating that these catalyst layers possess greater resistance to deformation.

From the tan δ curve shown in Figure 9c, the sample glass transition temperatures were determined. The peak of tan δ observed at the glass transition temperature indicates that the mobility of polymer chains increases as they undergo the transition from a glassy to a rubbery state. Therefore, as the degree of freedom increases, E″ also increases. As a result, the substance exhibits a decrease in rigidity and an increase in elasticity. Increased values of tan δ signify elevated energy dissipation capacity of the material. Conversely, reduced values of tan δ indicate greater elasticity of the membrane, which enables it to store energy rather than dissipate it. According to the findings, the Ag-Cu membrane exhibits the greatest Tg value in comparison with Cu-Cu and Cu-Ag. This results in an elevation of the stiffness of chain molecules, which might help to withstand a higher pressure in water treatment.

### 3.8. Dielectric Properties

**Figure 10:**
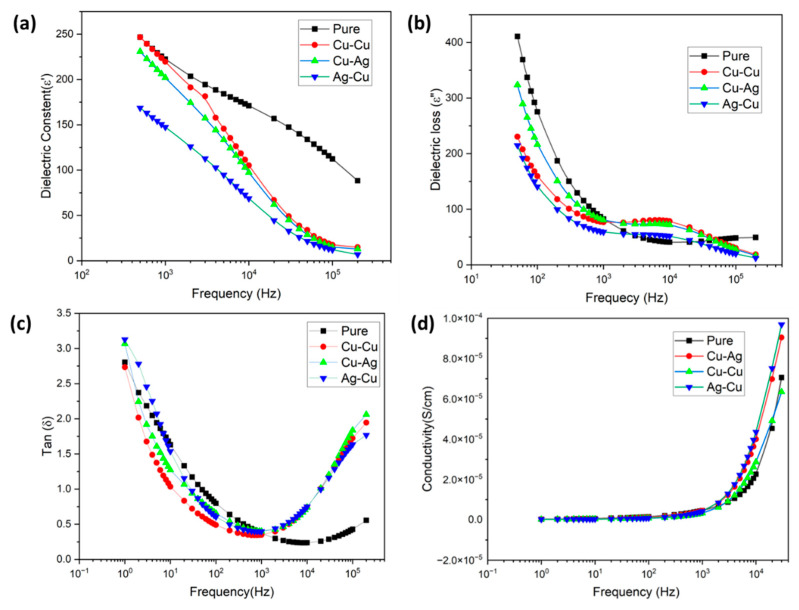
A study on the dielectric properties of membranes, both pure and with deposited metals.

The calculation of the dielectric constant is determined by utilizing the formula below.

$$\varepsilon_{r}=\frac{Cd}{A\varepsilon_{\text{0}}}$$

where *C* is capacitance, *d* is film thickness, *A* is electrode area, and *ε*_0_ = 8.854 × 10^−12^ F·m^−1^ is the free space permittivity. Figure 10a displays the dielectric constant (*ε*′) shift as a function of the logarithm of frequency for all the films.

The plot Figure 10a illustrates that the dielectric constant displays a larger value within the lower frequency spectrum, followed by a decline as the frequency rises across all films. The elevated value of the dielectric constant can be attributed to the phenomenon of the Maxwell–Wagner polarization. The genesis of this type of polarization can be traced back to the interfaces between insulators and conductors. As Nafion is highly proton conductive, it is evident that the dielectric constant of pure membranes is greater than that of metal-coated membranes. In the absence of an influx of electric charge via the electrode, the membrane space charges and dipoles are able to move freely at both low and high frequencies. However, accounting for the interaction between the applied electric field and the charges present in the surface results in the redistribution of ions within the film, leading to changes in the local electric potential and the formation of an electric double layer dropped the dielectric constant value by ~55% in Ag-Cu compared with pure membrane.

Similarly, Figure 10b illustrates a decrease in dielectric loss (ε″) with an increase in frequency. At low frequencies, polymer chains contain charge carriers with high mobility. In the presence of an electric field, the conductivity passage into the membrane is low, and the dissipation of energy is higher in pure membranes compared with Cu-Cu, Cu-Ag, and Ag-Cu membranes.

The dielectric loss in a material is typically quantified by the dielectric loss factor, also known as tangent delta (tan δ) (Figure 10c). The dielectric loss factor can be expressed in relation to the real and imaginary components of the dielectric constant.

$$\mathrm{Tan}\;\delta =\frac{\;\varepsilon \prime \prime }{\;\varepsilon \prime }$$

The primary sources of dielectric loss in materials are typically attributed to the conduction mechanism. As the surface conductivity surpasses the passage threshold, conductive networks are established with inner water molecules, leading to a low conduction loss. A positive correlation exists between the value of tan delta and the damping coefficient [28]. Consequently, membrane Ag-Cu had higher tan delta values than other samples and exhibited greater efficiency in effectively absorbing and dispersing energy. Which would be helpful for the triboelectric layer in energy generation. The following equation calculates conductivity,

$$\sigma =\frac{t}{RA}\;s\cdot {\mathrm{cm}}^{-1}$$

where *t* is the film thickness, *R* is the resistance, and *A* is the electrode area. From Figure 10d, it is clear that the conductivity of the membrane was increased to 1 × 10^−4^ through the electroless plating process on the surface of the film in Ag-Cu, which is enough to transport the voltage in nanogenerators.

## 4. Conclusions

The present research demonstrates that it exhibited several beneficial features and projected its distinctive behavior through the incorporation of Cu and Ag onto the PTFE-reinforced Nafion membrane. The following are the results that are favorable and attractive for practical applications.

- Enhanced mechanical properties were gained by metal ion deposition in addition to the reinforcement when compared with the unmodified membrane.
- The membranes that have been altered through a chemical deposition technique exhibit a more hydrophobic nature. This is due to the strong attraction between positively and negatively charged ions, which results in an adhesive nature and a 50% increase in the contact angle of Ag-Cu.
- Scratch test indicated that the adhesive strength between membrane and metal electrode was higher for Ag-Cu coated membrane.
- Increased crystallinity and phase transformation succeeded, and it would help in the generation of electrical energy.
- The Ag-Cu membrane demonstrates the highest Tg when compared with Cu-Cu and Cu-Ag. The observed outcome entails an increase in the rigidity of chain macromolecules, which could potentially enhance their ability to cope with elevated pressure during water purification processes.
- The successful achievement of improved conductivity outcomes was observed in specimens that underwent treatment in a reducing solution along with the presence of metal ions.

## Figures and Tables

**Table 1:** Chemical compositions for copper electroless plating process.

CuSO_4_·5H_2_O	EDTA·2Na	2,2-Dipyridine	HCHO	NaOH
40 g/L	35 g/L	0.05 g/L	25 mL/L	Until PH-12.5

**Table 2:** Mechanical properties of IPMC samples.

Membrane	Young’s Modulus (MPa)	Tensile Strength (MPa)	Elongation at Break (%)
**Cu-Cu**	779 ± 80	29.1 ± 8	67.3 ± 12
**Cu-Ag**	628 ± 65	23.5 ± 5	97.4 ± 7
**Ag-Cu**	470 ± 42	25.8 ± 2	158 ± 4

**Table 3:** Represent the adhesive strength and CoF values of all samples.

Membrane	Adhesive Strength (P) N/mm^2^	Max. Coefficient of Friction (CoF)
**Cu-Cu**	0.032	0.019
**Cu-Ag**	0.051	0.014
**Ag-Cu**	0.068	0.011

## Data Availability

The data presented in this study are available upon request from the corresponding author.

## References

[1] Maia B.A., Magalhães N., Cunha E., Braga M.H., Santos R.M., Correia N. (2022). Designing Versatile Polymers for Lithium-Ion Battery Applications: A Review. Polymers.

[2] Kim J., Park H., Yoon C. (2022). Advances in Biodegradable Soft Robots. Polymers.

[3] Alam M.W., Islam Bhat S., Al Qahtani H.S., Aamir M., Amin M.N., Farhan M., Aldabal S., Khan M.S., Jeelani I., Nawaz A. (2022). Recent Progress, Challenges, and Trends in Polymer-Based Sensors: A Review. Polymers.

[4] Hidalgo A.M., Murcia M.D. (2021). Membranes for Water and Wastewater Treatment. Membranes.

[5] Tanabe I., Imoto I., Okaue D., Imai M., Kumagai S., Makita T. (2021). Electronic excitation spectra of organic semiconductor/ionic liquid interface by electrochemical attenuated total reflectance spectroscopy. Commun. Chem..

[6] Hao M., Wang Y., Zhu Z., He Q., Zhu D., Luo M. (2019). A Compact Review of IPMC as Soft Actuator and Sensor: Current Trends, Challenges, and Potential Solutions from Our Recent Work. Front. Robot. AI.

[7] Porfiri M. (2019). Sensing mechanical deformation via ionic polymer metal composites: A primer. IEEE Instrum. Meas. Mag..

[8] Török B., Schäfer C., Kokel A., Török B., Schäfer C., Kokel A. (2022). Chapter 2—Solid catalysts for environmentally benign synthesis. Heterogeneous Catalysis in Sustainable Synthesis.

[9] He Q., Yin G., Vokoun D., Shen Q., Lu J., Liu X. (2022). Review on Improvement, Modeling, and Application of Ionic Polymer Metal Composite Artificial Muscle. J. Bionic Eng..

[10] Mayadevi T.S., Goo B.H., Paek S.Y., Choi O., Kim Y., Kwon O.J., Lee S.Y., Kim H.-J., Kim T.-H. (2022). Nafion composite membranes impregnated with polydopamine and poly (sulfonated dopamine) for high-performance proton exchange membranes. ACS Omega.

[11] Jain R.K., Datta S., Majumder S. (2012). Design and Control of an EMG Driven IPMC Based Artificial Muscle Finger. Computational Intelligence in Electromyography Analysis—A Perspective on Current Applications and Future Challenges.

[12] Dhapola P.S., Singh P.K., Bhattacharya B., Surana K., Mehra R., Gupta M., Singh A., Singh V., Sahoo N.G. (2018). Electrical, thermal, and dielectric studies of ionic liquid-based polymer electrolyte for photoelectrochemical device. High Perform. Polym..

[13] Tiwari R., Kim K.J. (2013). IPMC as a mechanoelectric energy harvester: Tailored properties. Smart Mater. Struct..

[14] Ghosh S. (2019). Electroless copper deposition: A critical review. Thin Solid Films.

[15] Huhtamäki T., Tian X., Korhonen J.T., Ras R.H.A. (2018). Surface-wetting characterization using contact-angle measurements. Nat. Protoc..

[16] Rajesh K., Crasta V., Kumar N.B.R., Shetty G., Rekha P.D. (2019). Structural, optical, mechanical and dielectric properties of titanium dioxide doped PVA/PVP nanocomposite. J. Polym. Res..

[17] Kanamarlapudi S.L.R.K., Chintalpudi V.K., Muddada S. (2018). Application of Biosorption for Removal of Heavy Metals from Wastewater. Biosorption.

[18] Yu S., Zhu Z., Zhou M., Yu H., Kang G., Cao Y. (2021). Fabrication and characterization of a novel Nafion-PTFE composite hollow fiber membrane. J. Appl. Polym. Sci..

[19] Tsiaxerli A., Karagianni A., Ouranidis A., Kachrimanis K. (2021). Polyelectrolyte Matrices in the Modulation of Intermolecular Electrostatic Interactions for Amorphous Solid Dispersions: A Comprehensive Review. Pharmaceutics.

[20] Yesaswi C.S., Sreekanth P.R. (2020). Evaluation of dynamic mechanical properties of teflon fabric reinforced artificial muscle material. Mater. Today Proc..

[21] Ribeiro C., Costa C.M., Correia D.M., Nunes-Pereira J., Oliveira J., Martins P., Correia D.M., Correia V., Ribeiro C., Pedro Martins P.M., Ameduri B., Fomin S. (2020). Chapter 1—Electroactive poly (vinylidene fluoride)-based materials: Recent progress, challenges, and opportunities. Fascinating Fluoropolymers and Their Applications.

[22] Kusoglu A., Weber A.Z. (2017). New Insights into Perfluorinated Sulfonic-Acid Ionomers. Chem. Rev..

[23] Malaki M., Fadaei Tehrani A., Niroumand B., Gupta M. (2021). Wettability in Metal Matrix Composites. Metals.

[24] Qasem N.A.A., Mohammed R.H., Lawal D.U. (2021). Removal of heavy metal ions from wastewater: A comprehensive and critical review. npj Clean Water.

[25] Yang L., Zhang D., Zhang X., Tian A. (2021). Electroless copper deposition and interface characteristics of ionic electroactive polymer. J. Mater. Res. Technol..

[26] Wang X., Xu P., Han R., Ren J., Li L., Han N., Feng X., Zhu J. (2019). A review on the mechanical properties for thin film and block structure characterised by using nanoscratch test. Nanotechnol. Rev..

[27] Yesaswi C.S., Sreekanth P.S.R. (2022). Characterisation of Silver-coated Teflon fabric-reinforced Nafion ionic polymer metal composite with carbon nanotubes and graphene nanoparticles. Iran Polym. J..

[28] Billah S.M., Jafar Mazumder M., Sheardown H., Al-Ahmed A. (2018). Dielectric Polymers. Polymers and Polymeric Composites.

